# A flexible theoretical representation for the temporal dynamics of structured populations as paths on polytope complexes

**DOI:** 10.1007/s00285-014-0841-4

**Published:** 2014-10-12

**Authors:** Armando J. R. Amaris, Murray P. Cox

**Affiliations:** Institute of Fundamental Sciences, Massey University, Palmerston North, New Zealand

**Keywords:** Migration, Population structure, Population genetics, Polytope complex, Circle patterns, 92D25, 52B99, 52C26

## Abstract

We present a new theoretical framework to represent the dynamics of structured populations through time and across geographic space. We show (i) that the mechanisms by which populations evolve lead to combinatorial structures, and (ii) that measures of gene flow and geographical structure lead to linear systems. These characteristics determine two polytope complexes that encodes all feasible migration scenarios. Analysis of these polytope complexes demonstrates how systems of structured populations can be classified consistently, and how population histories can be represented as paths on a concrete mathematical space, which in turn promises to simplify the search space required for reconstructing past migration processes from population genetic data.

## Introduction

Many demographic factors, including movements of both individuals and entire populations, can alter the nature of linkages between populations over time. Representing the diverse forms of these temporal dynamics requires a flexible theoretical framework that is consistent with longer-term goals of reconstructing the history of structured populations (“metapopulations”) from population genetic data. We show that the key characteristics of these migration dynamics—the intensity of migration, pathways of migration and the spatial distribution of populations—give rise to various mathematical structures that allow us to view populations and migration between them as geometric objects, and their dynamics as pathways on a polytope complex $\mathfrak {K}$. Here, we construct such a framework and propose it as a powerful mathematical foundation for studying complex metapopulation dynamics.

To illustrate the basic ideas that motivate our work, consider the ‘Out of Africa’ model, in which anatomically modern humans originated in a small region of Africa, leaving $\sim$50,000 years ago to begin the settlement of Eurasia. On reaching the Middle East, the population divided, one group moving towards Europe, while the other continued on to Asia. Subsequent splitting (and joining) events produced the distribution of human populations observed today.

**Fig. 1:**
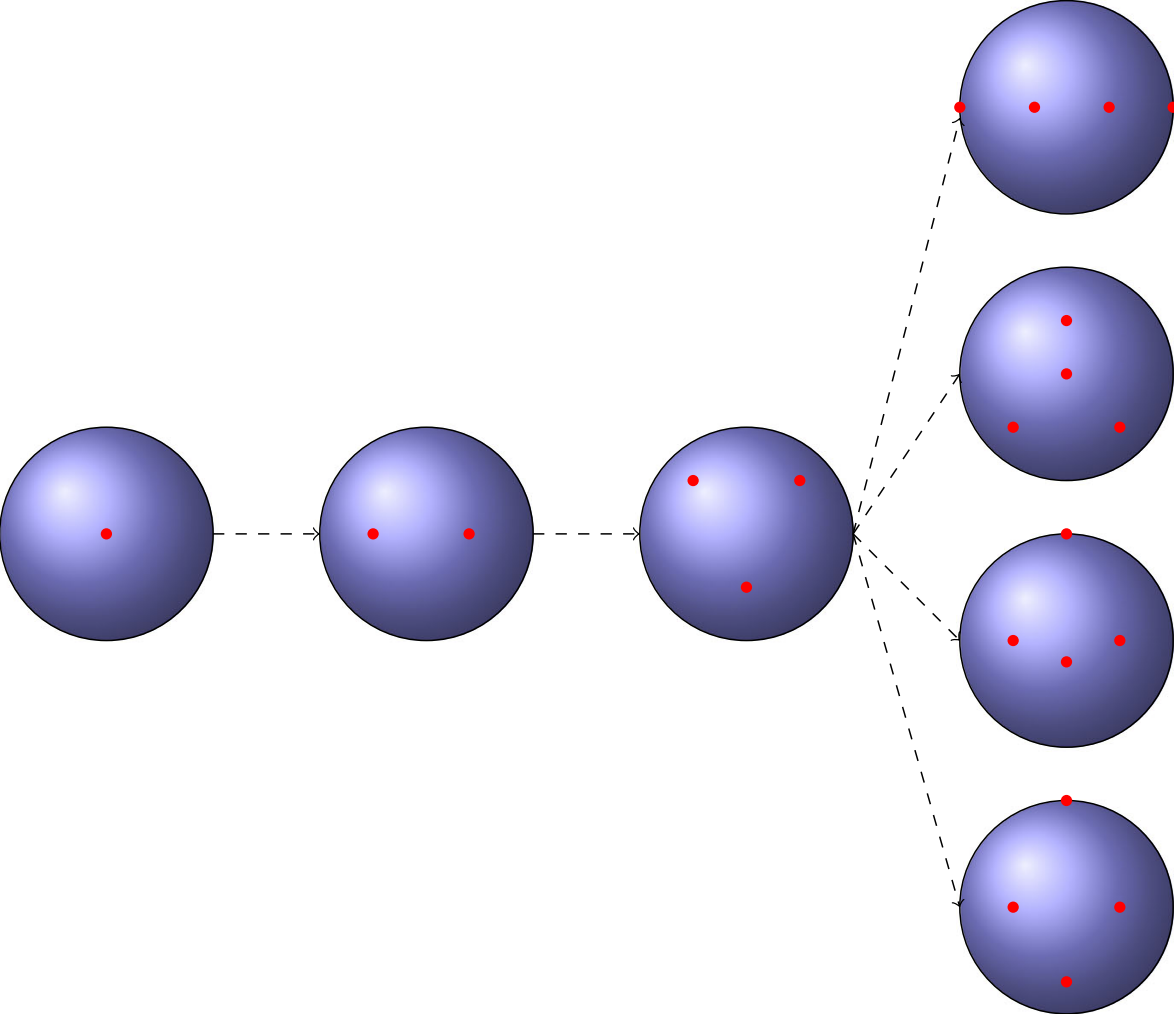
*Each sphere* represents the planet Earth, on which a very simple migration progression has been illustrated. Starting from a single population (*far left*), mimicking the “Out of Africa” model, the population subsequently splits into two, and then three populations, each of which allows only one possible geometric arrangement. On splitting into four populations, four possible geometrical arrangements are now possible. The combinatorics of possible geometrical arrangements continues to increase with the number of populations

A model of this type can be stated more formally. If we denote the number of populations at time $t$ by $N(t)$, then initially $N(t_0)=1$. At the time of the first split $t_2$, $N(t_2)=2$; at $t_3, N(t_3)=3$; and so forth. Note that these three populations are necessarily all located on the circumference of a hypothetical sphere—here, an individual can only migrate to its immediate neighbors, although the probability of migrating to any given neighbor might be large or small. As the number of populations increases to four, the possible geometric patterns produced are no longer unique. As shown by geometric-combinatorial arguments, the relative locations of the populations can instead adopt a range of pattern settings (represented in Fig. 1).

This basic summary raises the question of whether there is a general description of the space of all possible migration scenarios, within which populations can move, split and merge through time. This is the first question that we address. Subsequently, such a pure combinatorial-geometric analysis based solely on the location of populations is complemented by considering measures of migration between populations. It is assumed that migration occurs via neighboring demes, which was typical during much of prehistory when human mobility, constrained by geography and available technologies, was largely restricted to continuous paths on land or by water.

Considering the theoretical mathematical structures that arise as part of this theory is beneficial for a number of reasons: (i) it allows the relationships between alternative migration scenarios to be quantified; (ii) it can be used to test the results of current models and software that describe migration among populations; and (iii) it could ultimately be employed in a statistical inference setting to reconstruct likely migration histories based on genetic information. The theory allows the number of populations to change through time, and it facilitates comparison between different metapopulation systems. However, we will begin by considering the simplest state: a fixed number of populations, each of which is represented by a point, with migration indicated by edges. Weights assigned to these edges represent a measure of both population and individual migration. The graph determined by these points and edges is also defined to capture spatial factors, such as the physical distance between populations and the possible presence of any intervening geographical barriers, such as mountain ranges or water crossings.

The fact that populations interact on a planet—a three-dimensional sphere—might be obvious, but is worth emphasizing because it implies certain constraints on population structure. For instance, when we consider migration among a fixed number of populations, the interacting populations can only form a limited number of graph structures determined by a sphere with $n$ marked points. In addition, if a group of populations has limited mobility over geographical space (i.e., movement is not arbitrarily free), a simple but important observation is that every migration path from a given location $L$ to a chosen destination $D$ should cross the boundary of a region containing all points that are closer to $L$ than to any other population. In other words, points $L$ and $D$ comprise the cells of a Voronoi tessellation. The following paragraph defines such a Voronoi cell, which forms a fundamental basis of our migration theory.

### **Definition 1**

Given a set of points $P$ on a surface $S$ with metric $d$, the Voronoi cell associated with $i \in S$ is the set of all points $j$ in $S$ that satisfy $d(i,j) \le d(i',j)$ for all $i' \in P$. The Voronoi diagram $G$ associated with $P$ is the set of all Voronoi cell boundaries. Further, we will say that $P$ and $d$ determine a Voronoi cell decomposition of $S$.

Observe that even though the locations of a set of points are needed to compute a Voronoi diagram, this is not overly restrictive. First, populations are usually restricted geographically to well defined regions, with these regions typically well separated. For most biological species, it is sensible to define some form of population grouping, to which a geographical center can then be assigned. Second, our model only uses the center of a population as a way to define boundaries between regions. The individuals themselves could be dispersed within this region. Our model holds as long as the population has some form of geographical center (regardless whether individuals actually live at that point).

In the case of a geographically static set of populations (i.e., the populations themselves do not move), each population belongs to a Voronoi cell whose shape is invariant through time. However, even in the special (and biologically unrealistic) case of geographical stasis, the chosen distance measure (“migration”) linking neighboring cells may potentially change through the movement of individuals between populations. In another special case, populations can change their geographical location, while maintaining the same level of migration between them (e.g., as is the case for seasonal nomadic populations). In more general settings, population movements and individual migration are interrelated, and the Voronoi cell tessellation must represent both characteristics jointly. With this general framework in mind, we define the concept of a migration pattern in the following section.

Voronoi diagrams and related theory have been applied to a wide range of scientific problems (see Okabe et al. 2000 for a survey of applications). Their use in biology has an especially long history. For instance, Voronoi diagrams have been used to analyze the geometric structure of biological molecules, to cluster biological data, and to estimate the volume and surface of interphase chromosomes (Lee and Richard 1971; Ban et al. 2004; Kim 2004). However, we are not aware of prior applications to migration theory or analysis, especially using combinatorics.

### The space of migration patterns

**Fig. 2:**
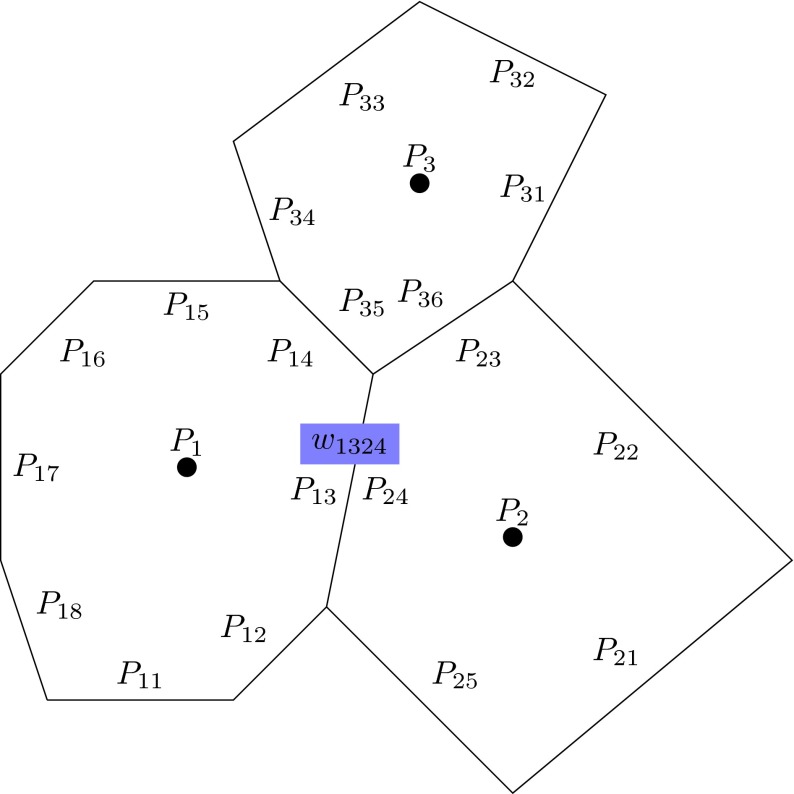
Three polygons representing Voronoi cells containing populations $P_1$, $P_2$ and $P_3$. The boundary edge between the cells containing $P_{1}$ and $P_2$ has been assigned a weight $w_{1324}$, which is determined by the rate of migration between $P_{1}$ and $P_2$. Weights are assigned in a similar way to all edges in the Voronoi diagram

Consider a set $P$ of $n$ populations across a geographical region, where each population is represented by a point. At any given time, there is a migration pattern associated with $P$, which can be represented by a Voronoi diagram determined by $P$ with gene flow passing across each of its edges. If the position of a population changes with time, the Voronoi cell decomposition determined by $P$ may also change. However, fixed locations of populations do not necessarily imply a fixed migration pattern since migration patterns can also change through variation in levels of individual migration. Consequently, the graph nature of migration—both the movement of entire populations and the movement of individuals between populations—should ideally be represented within the same mathematical structure. We define the intensity of migration between two populations through edge $e_{P_iP_j}$ linking populations $P_i$ and $P_j$ as the weight $w_{P_iP_j}$, some number that at present we scale to the interval $(0,1)$ (see Fig. 2). We can also assign the weight $w_{P_iP_j}$ to the corresponding Voronoi boundary, so that it is possible to pass from a weighted Voronoi diagram to a weighted graph with populations as vertices (i.e., the dual graph of the Voronoi diagram). Initially, we will assume that the sum of migration into and out of each population is constant, although we will discuss later how this condition can be removed.

We define migration patterns as being the same if their corresponding graphs are equivalent under homeomorphism^1^ and their corresponding edges^2^ have the same weights. However, as we specifically consider migration on a sphere, we include a set of numbers $\varTheta =\{\theta _i\}_{i \in I}$ in our definition, where each $\theta _i$ can be considered an intersection angle between two circles that belong to a family of circles uniquely associated with the migration pattern graph. $\varTheta$ captures the geographical dimension of a migration pattern, while allowing migration patterns to be identified uniquely among different metapopulations, which may be widely separated geographically, but possess the same underlying geographical structure. In addition, we also include a set of weights $W$ in our definition, which represents a measure of gene flow between populations,^3^ and a set $T$, which measures the total flow crossing each population.

#### **Definition 2**

The migration pattern of a set $P=\{P_1,P_2,\ldots ,P_n\}$ of $n$ populations is a 4-tuple $M=(G,\varTheta ,W,T)$, where $G$ is the Voronoi diagram on the two dimensional sphere $S^2$ determined by the set of geographical coordinates of $P$; $W=\{w_e\}_{e \in E(G)}$ is a set of positive numbers such that $0 < w_e < 1$ and the sum of weights $w_e$ on the face of $G$ corresponding to $P_i$ is $T_i$, where $T=\{T_1,T_2,\ldots ,T_n\}$; and $\varTheta =\{\theta _e\}_{e \in E(G)}$ is a set of positive numbers.

We call $T$ the set of total loads of $M$ (or its vertex weights). For a set of populations in geographical space, it can be shown that a collection of circles exists that contains, for all populations in their boundary, the set of weights $\varTheta =\{\theta _i\}_{i \in I}$ corresponding to the set of interior angles associated with this collection of circles. More details follow on this point in Sect. 2.2.

We declare two migration patterns to be identical if their $W$ and $\varTheta$ weighted graphs are equivalent. Further, we call the collection of all migration patterns, for a system of $n$ populations with total weight $T$, the space of migration patterns $MS(n,T)$. The migration graph of $M$ will be called $G$. We assume that the ends of every edge of a migration graph are different, which implies that a migration graph has at least two vertices (i.e., we do not consider the theoretical, but biologically unrealistic, case of a population that is completely enclosed by another population). Finally, the collection of all migration graphs of $MS(n,T)$ will be called the space of combinatorial structures of $MS(n,T)$, denoted by $CombMS(n)$.^4^

Note that our model could be modified to use $\varTheta$ as weights on a directed graph, dual of the Voronoi diagram with centers being the populations under study. We opt not to do so because, in practice, computing the direction of migration is difficult (Hey 2010), while nondirectional measures like $F_{ST}$ are more amenable to calculation (Cox and Hammer 2010). We prefer to model non-directed graphs to provide flexibility of choice around measures of migration. Nevertheless, implementing a directed graph structure is a natural extension of this research, and as directional measures of migration improve, a directed graph structure will become an increasingly worthwhile pursuit.

### Paper outline

In the introduction, we defined the space of migration patterns $MS(n,T)$ as a natural setting to study the movement of, and individual gene flow between, a fixed number of populations that interact across geographical space. In the following sections, we will explore the rich mathematical nature of $MS(n,T)$ by studying its combinatorial and graph structure. In the subsection on linear systems and polytopes, we will show that two linear systems of equations/inequalities can be associated with each migration graph, and indicate how our approach leads to two Euclidean polytope complexes that encode all of the information provided by the migration pattern. We will then show how the evolution of a metapopulation system through time can be viewed as a path on a specific polytope complex when the number of populations is fixed, and later extend this finding to more general cases where populations can split and merge. We then provide a real world example showing how this mathematical framework can be applied. The paper concludes with a general discussion of the theory. Finally, we provide an appendix with mathematical proofs required to support all of the new mathematical ideas introduced in this work.

## Methods

### The combinatorics of migration patterns

In this subsection, we consider only the space of combinatorial structures $CombMS(n)$ for a fixed number of populations $n$. Hence, we assume that the number of cells in the Voronoi cell decomposition of the sphere is also $n$. The number of edges may vary, but the degree of the vertex—the number of edges incident to it—cannot be less than three.

A basic result that relates the number of vertices $V$, the number of faces $F$ and the number of edges $E$ of a cell decomposition of the sphere is Euler’s characteristic formula (Flegg 2001; Richeson 2008):

1 $$\begin{aligned} V+F-E=2 \end{aligned}$$

**Fig. 3:**
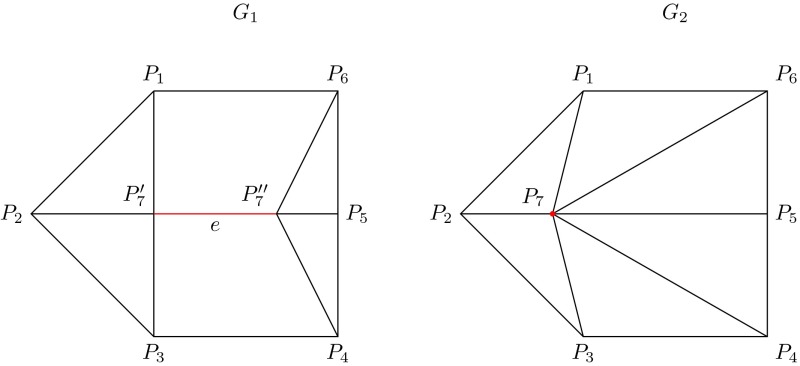
The graph $G_1$ (*left*) can produce the graph $G_2$ (*right*) by a contraction move on edge $e$ (*red*). Equivalently, the graph $G_1$ can be obtained from the graph $G_2$ by an expansion move on the internal vertex $P_7$, which is determined by the vertices $P_6$, $P_1$, $P_3$ and $P_4$

Three edge transformations facilitate the description of migration graphs in the space $CombMS(n)$. The first transformation is called a *contraction move*, a transformation that when applied to an edge $e$ of a migration graph, continuously reduces its length to zero, in such a way that all other edges are topologically preserved (Fig. 3). A contraction move on an edge produces a new graph whose number of edges is reduced by one.

The second transformation is called an *expansion move*. It creates a new edge $e'$ by splitting a vertex $P$, of valence greater than 3, into two vertices $P_1$ and $P_2$ such that $e$ connects $P_1$ and $P_2$, while all edges formerly incident to $P$ remain incident to either $P_1$ or $P_2$. Observe that the valences of the vertices $P$, $P_1$ and $P_2$ are related by:

2 $$\begin{aligned} valence(P_1)+valence(P_2)=valence(P)+2 \end{aligned}$$

**Fig. 4:**
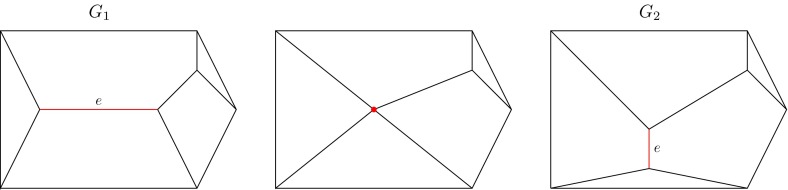
The graphs $G_1$ (*left*) and $G_2$ (*right*) are connected by a Whitehead move on edge $e$ (*red*). The middle graph is an intermediate step where the edge $e$ has been collapsed to a single point

The third transformation is called a *Whitehead move*. We illustrate a Whitehead move on edge $e$ of graph $G_1$ in Fig. 4. It is convenient to imagine edge $e$ as being continuously contracted until it collapses to a single point, as represented in the central graph. The process continues by expanding that point into a new vertical edge, also denoted by $e$, to produce graph $G_2$. In practice, a Whitehead move changes $G_1$ to $G_2$ in a single step. Whitehead moves have been used to describe the combinatorics of moduli spaces (see Amaris 2007; Kasra and Tao 2013). They have a central role in our theory because Whitehead moves connect all cubic migration patterns in $CombMS(n)$, as described in Proposition 1 (see below). This in turn simplifies the analysis and description of general migration patterns since they can be viewed as being imbedded in a cubic migration pattern.

#### **Proposition 1**

If $G_1$ and $G_2$ are two cubic migration graphs in $CombMS(n)$, then a sequence of Whitehead moves $Wh_{e_1},Wh_{e_2}, \ldots , Wh_{e_k}$ exists such that

$$\begin{aligned} Wh_{e_k} \circ Wh_{e_{k-1}} \circ \ldots \circ Wh_{e_1} (G_1)=G_2 \end{aligned}$$

A proof for this proposition is given in the Appendix.

Using the edge transformations listed above, we can now describe $CombMS(n)$ as follows:

#### **Proposition 2**

All migration graphs in $CombMS(n)$ are connected by a sequence of contraction moves to a cubic graph. More exactly, for every graph $G$ in $CombMS(n)$, there exists a graph $\widehat{G}$ in $CombMS(n)_0$, a unique number $k$ and a sequence of contraction moves $c_1,c_2, \ldots , c_k$ such that

$$G=c_k \circ c_{k-1} \circ \ldots \circ c_{1}(\widehat{G})$$

#### *Proof*

**Fig. 5:**
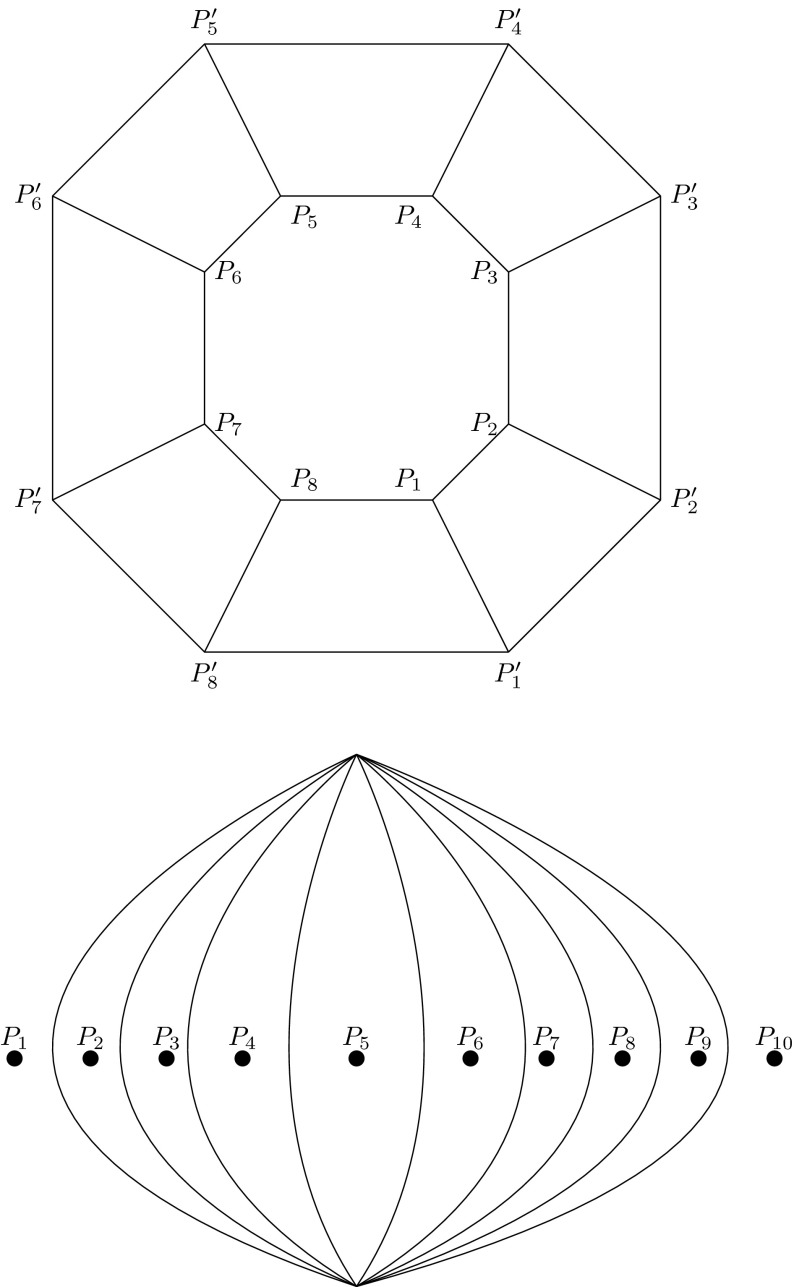
The *upper graph* $CL_{10}$ can generate all cubic graphs of $MS_{10}$ by Whitehead moves, which in turn can generate the whole $MS_{10}$ by contraction moves. The *lower graph* $C_{10}$ is a representation of the migration pattern for ten populations located on a geographical great circle. $C_{10}$ can generate all the graphs in $MS_{10}$ by a combination of expansion or contraction moves

This argument can be proved by showing that any cubic graph embedded in a two-dimensional sphere with $n > 4$ faces can be obtained from the circular wheel graph $CL_n$ (Fig. 5) by Whitehead moves, where $CL_n$ is constructed by taking two concentric copies of a regular polygon of $m=n-2$; for instance, with vertices $P_1,P_2,\ldots , P_m$ and $P'_1,P'_2,\ldots , P'_m$ and adding all edges of the form $P_iP'_i$, $i \in \{1,2,\ldots ,m\}$ (see Fig. 5 for the case of $n=10$).

As a consequence of this proposition, we can prove that all graphs in $CombMS(n)$ are connected by contraction or expansion moves.

#### **Proposition 3**

For every pair of graphs $(G_1,G_2)$ in $CombMS(n)$, a sequence of contraction or expansion moves $m_1,m_2, \ldots , m_k$ exists such that

$$G_2=m_k \circ m_{k-1} \circ \cdots \circ m_{1}(\widehat{G_1})$$

Proposition 1 can be viewed as a description of $CombMS(n)_0$, the upper layer of $CombMS(n)$. This in turn can be considered as a generating set for $CombMS(n)$, in the sense that all graphs in $CombMS(n)$ can be obtained from $CombMS(n)_0$ by contraction moves. In this proposition, the graph $\widehat{G}$ is not unique. However, the uniqueness of $k$ allows us to define deeper layers of the structure of $CombMS(n)$. With this in mind, we define the depth of a migration graph $G$ as the number of contraction moves needed to obtain $G$ from a cubic graph. Further, we define the *layer* or *strata* of $CombMS(n)$, $CombMS(n)_k$, as the collection of all migration patterns at the same depth $k$. At the deepest level of $CombMS(n)$, there is a single graph $C_n$ with exactly two vertices and $n$ edges. This graph arises naturally when considering a set of $n$ populations that lie on a spherical geodesic (with respect to the great circle metric), but as noted above, this structure also has purely mathematical utility. Figure 5 shows the example $C_{10}$.

Note that Euler’s characteristic formula (Eq. 1) implies that every migration graph in $CombMS(n)_0$ has $n$ faces, $2n-4$ vertices and $3n-6$ edges. Hence, $k=2n-5$ contraction moves are needed to reach the deepest layer of $CombMS(n)$. The number of vertices and edges at each layer of $CombMS(n)$ can easily be computed, which implies the following proposition:

#### **Proposition 4**

$$\begin{aligned} CombMS(n)=\cup _{k=0}^{2n-5}{CombMS(n)_k} \end{aligned}$$

The propositions above provide several alternative perspectives of $CombMS(n)$. An additional perspective is to view $CombMS(n)$ as being generated from the graph $C_n$, defined above, via transformations provided by expansion or contraction moves. The application of single expansion moves to $C_n$ will produce a second generation of graphs at a higher level, which can in turn be used as seeds for a third generation of graphs. As new generations are expanded, not all graphs in $CombMS(n)$ are necessarily obtained (see Amaris 2007, page 62). Indeed, a combination of expansion and contraction moves are required to move through the whole combinatorial space. In other words, graphs exist in $CombMS(n)$ that cannot be obtained solely by a sequence of expansion moves from $C_n$. This third perspective on $CombMS(n)$, which is a consequence of Proposition 2, is summarized in the following proposition:

#### **Proposition 5**

For every graph $G$ in $CombMS(n)$, a sequence of expansion and/or contraction moves $m_1,m_2, \ldots , m_k$ exists such that

$$G=m_k \circ m_{k-1} \circ \cdots \circ m_{1}(C_n)$$

Jointly, the perspectives given by the propositions above can be used to obtain an explicit description of $CombMS(n)$. For example, with just two populations, $CombMS(2)$ comprises a single migration graph: a simple loop. In the three population case, $CombMS(3)$ contains only one graph with two vertices and three edges. In the four population case, $CombMS(4)$ has four possible migration graphs: two cubic graphs, one graph with three vertices, and one graph with two vertices—i.e., $C_4$ (see Fig. 1 for a graphical representation). To explicitly enumerate graphs, it is important to remember that two equivalent graphs in $CombMS(n)$ are considered identical.

### Graph duality

**Fig. 6:**
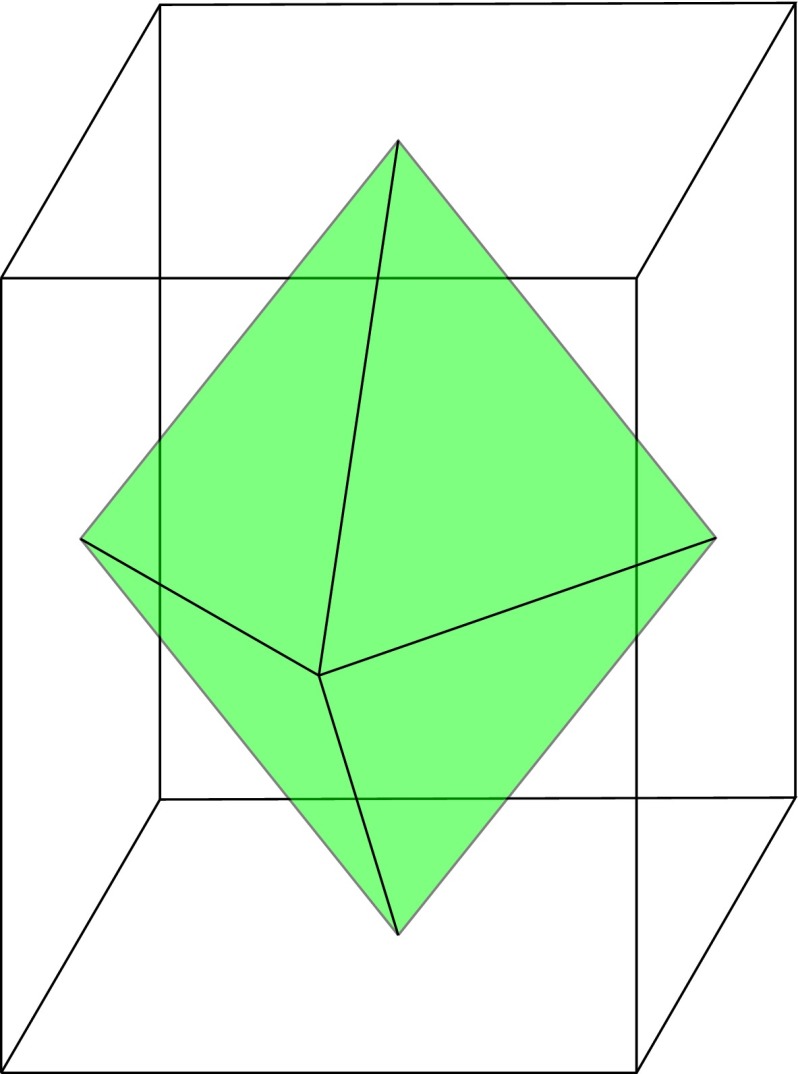
The graph $C_6$ (*clear*) and its corresponding dual graph $O_8$ (*green*), as determined by the edges of a cube and an octahedron, respectively

Any graphs that can be embedded in the two-dimensional sphere can be related by a graph duality relationship. To explain this concept, consider a graph $G$. To build the dual graph $G^{\circ }$ of $G$, simply mark each face of $G$ (e.g., by drawing a single point in its interior), and then for each edge of $G$, draw a new edge joining the two marked points on the adjacent faces. For example, in Fig. 6, we mark a point on each of the six faces of a cube. This produces the vertices of the graph $O_8$ (green), which is the dual graph of $C_6$ (clear).

Graph duality allows us to move to and from equivalent graph representations of the same phenomenon—in our case, a migration pattern. Each view emphasizes different aspects of the system. For instance, we can determine which population is closest to an individual just by determining the Voronoi cell in which that individual is located. By using its dual graph, we can potentially identify whether an individual came from a given population by direct migration simply by considering whether vertices representing the source and sink populations are connected by an edge, assuming the sink and source populations are known. This concept of graph duality can easily be extended to the concept of duality for the entire cell decomposition of the sphere:

#### **Definition 3**

Given a graph $G$, we denote the dual graph by $G^{\circ }$. If $G$ is the Voronoi diagram determined by a set of points $P$, then $G^{\circ }$ is the Delaunay graph determined by $P$. Similarly, the dual of a cell decomposition of the sphere $D$ is denoted by $D^{\circ }$.

**Fig. 7:**
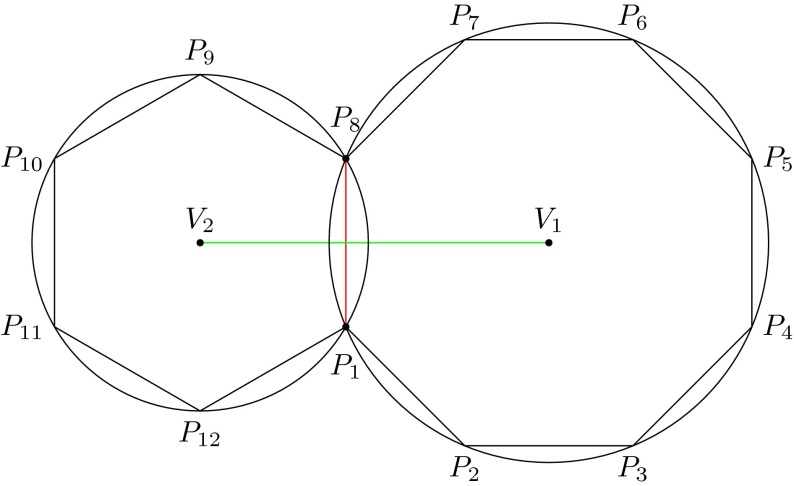
Two Delaunay cells with vertices $P_1,P_2,\ldots , P_8$ and $P_8, P_9,\ldots , P_{12},P_1,P_8$ (e.g., possibly representing populations). The centers $V_1$ and $V_2$ of these cells are vertices in the Voronoi cell decomposition determined by these populations. The edge $e^{\circ }_i$ joining $P_1$ and $P_8$ (*red*) is assigned the weight $\theta _i$, which can be interpreted geometrically as the inner angle between the circles with centers $V_1$ and $V_2$ (the measure of the angle $V_2P_8V_1$). The edge joining $V_1$ and $V_2$ (*green*) is assigned the weight $1-\theta _i$

Interestingly, it is possible to associate a set of circles—the collection of circles that have no vertex in their interior—to the dual graph of a Voronoi cell decomposition (see Springborn 2003). For example, Fig. 7 shows a partial view of a Delaunay cell decomposition determined by a set of points $P$, where $V_1V_2$ is one edge of the Voronoi cell decomposition determined by $P$, and the Delaunay cells with centers $V_1$ and $V_2$ are circumscribed by the given circles. More generally, if $D$ is a Voronoi cell decomposition, with dual cell decomposition $D^{\circ }$, it can be proved that each cell $R$ in $D$ is circumscribed by a circumference (as occurs in Fig. 7). Further, we can assign the interior angle $\theta _j$ to the edge $e$ of $G^{\circ }$, where an instance of an interior angle is given by the angle $V_2P_8V_1$ in Fig. 7. In this setting, it can be asked whether a circle pattern exists whose intersection angles correspond to the given set of values $(\theta _j)$. Rivin (1996) provided a solution to this problem. In our definition of a migration pattern, the parameter set $\varTheta$ should be understood as interior intersection angles in spherical geometry.

#### **Theorem 1**

(Rivin’s theorem) Let $\varSigma$ be a strongly regular cell decomposition of the sphere and let an angle $\theta _e$ with $0<\theta _e$ given for every edge of $\varSigma$. Let $\varSigma ^*$ be the dual decomposition of $\varSigma$, and for each edge $e$ of $\varSigma$, denote the dual edge of $\varSigma ^*$ by $e^*$.

A Delaunay pattern corresponding to $\varSigma$ with exterior angles $\theta _e$ exists if and only if the following conditions are satisfied:

1. If some edges $e^*_1,\ldots , e^*_n$ form a boundary face of $\varSigma ^*$, then
  $$\begin{aligned} \varSigma \theta _{e_j}=2\pi \end{aligned}$$
2. If some edges $e^*_1,\ldots , e^*_n$ form a closed path of $\varSigma ^*$, which is not the boundary of a face, then
  $$\begin{aligned} \varSigma \theta _{e_j}>2\pi \end{aligned}$$

If the circle pattern exists, then it is unique under a Möbius transformation of the sphere.

Note that Rivin’s theorem gives necessary and sufficient conditions for the existence of a Delaunay pattern with inner circle intersection angles provided by $\varTheta$. If a migration pattern is known, $\varTheta$ should satisfy conditions 1 and 2 above. Conversely, if a graph is drawn on a sphere with well defined cells (‘regions’) and a positive number is chosen for each of its edges, the corresponding dual graph is a migration pattern for the chosen weights if the conditions above are satisfied. Then, as we shall see in the following section, Rivin’s theorem and the combinatorial knowledge of migration patterns allows us to systematically generate all circle patterns. We are only concerned with Voronoi cell decompositions of the sphere that are strongly regular (for more details, see Springborn 2003). The conditions on the weights $w_e$ given in the definition of migration patterns above are independent of Rivin’s theorem. They instead arise as a mechanism to let us model migration among populations, but the specific computation of these weights and their precise interpretation must be specified in any given application. In particular, we favor commonly used indirect measures of migration (Beerli 1998), among which can be classified: (i) simple estimators based on allele frequencies (Michalakis and Excoffier 1996; ii) maximum likelihood estimators based on allele frequencies (Rannala and Hartigan 1996); and (iii) estimators based on genealogies of the sample (Wakeley 1998). We do not know whether the different measures $w_e$ and $w'_e$ should be related, but this seems unlikely at the level of generality that we are considering here.

### Linear systems for metapopulation analysis

For a system of populations (a ‘metapopulation’), a specific migration pattern can be determined whenever information is known about the location of populations, as well as migration between them, at a specific time. While this information can be obtained for many real populations today, the dynamics of these populations through time is unlikely to be known. However, an analysis of migration is still possible in many instances because basic features, such as the number of populations, impose important mathematical constraints on the evolution of the system. Indeed, the combinatorial analysis of $CombMS(n)$ has already revealed several such constraints. Moreover, Rivin’s theorem can be interpreted as a set of equalities and inequalities that, given necessary and sufficient conditions, determine a system of linear inequalities whose solutions lie on a polytope that is associated uniquely with the system under study.

Changing our focus to levels of individual migration across the system, further constraints are considered to provide a better description of migration patterns as dynamic entities that can change through time. Consider a given migration graph $G$ with a chosen edge labeling,^5^ and its dual graph $G^{\circ }$ with edges marked using the convention that pairs of dual edges have the same label. The intensity of migration between two populations joined by an edge in $G^{\circ }$ is represented by the variable $x_i$. Hence, we assign the linear system $L^{\circ }(G,T)$ to $G$ and $T$ as:

1. $$0<x_i<1$$
2. $\sum _{j \in E(P_i)}x_j=T_i$, for each vertex $P_i \in V(G^{\circ })$, where $E(P_i)$ and $V(G^{\circ })$ are the set of edges incident to $P_i$ and the set of vertices of $G^{\circ }$, respectively.

Note that the first constraint restricts each solution to lie in the interior of the unit hypercube of dimension $m$ (the number of edges of $G$), while the second constraint assigns a facet of a polytope contained in $[0,1]^m$ to each face of $G$. This allows us to assign a polytope to the family of all migration patterns associated with the same graph by relaxing the strict inequalities $<$ to $\le$. In many cases, the two polytopes that we have described can be obtained explicitly (e.g., by using the geometry software package, *Convex*; Franz 2000).

Although we could associate a non-empty polytope with the genetic system above, an abstract graph that can be embedded in the sphere is not necessarily realizable in spherical geometry. Further, spherical realizability for a given abstract graph does not imply realizability of the genetic linear system associated with the same graph. Important properties of the families of a polytope associated with migration patterns will be considered in the next section, which considers the underlying combinatorics of $CombMS(n)$. Our result links this new theory of migration patterns to polytope theory, which is a strong branch of pure and applied mathematics (see Ball 1997; Bayer and Lee 1993; Bokowski and Sturmfels 1989; Gruber and Wills 1993; Grünbaum et al. 2003; Ziegler 1994).

### Population histories as paths on $\mathfrak {K}$

The migration pattern associated with a metapopulation $M(t)=(G(t),\varTheta (t), W(t),T(t))$ is likely to change through time. However, $M(t)$ should change relatively slowly for processes that occur over long time scales. This implies that there are periods of time when the graph $G(t)$ is invariant. Thus, for a period of time $I$ when $G(t)$ is fixed, the polytope pair $K=(K_{[1]}(t),K_{[2]}(t))$ associated with $M(t)$ is also fixed. In this way, $M$ is a curve in $K(t)$—a curve in polytope $K_{[1]}(t)$ and a curve in polytope $K_{[2]}(t)$ that describes changes in the geographical arrangement of populations and gene flow between them. However, since the analysis is similar for both polytopes in most cases, we will describe $K$ as a single polytope when describing common properties.

The evolution of a set of populations can be described as a path in $K$ for some $t \in I$. Further, most systems will eventually undergo a change in $G(t)$, caused by a contraction or expansion move, and hence, the polytope $K(t)$ will also change. More generally still, a collection of polytopes appears in this process, and the structure of this collection of polytopes mirrors the combinatorial structure $CombMS(n)$.

To describe this new structure, we first consider $CombMS(n)_0$, an arbitrary cubic graph with labeling $l$, and extend its labeling to the whole stratum $CombMS(n)_0$ while keeping all labels invariant during any Whitehead moves.^6^ Since each labeled graph has an associated polytope, a polytope complex can be constructed that depends only on the initial labeled graph.

**Fig. 8:**
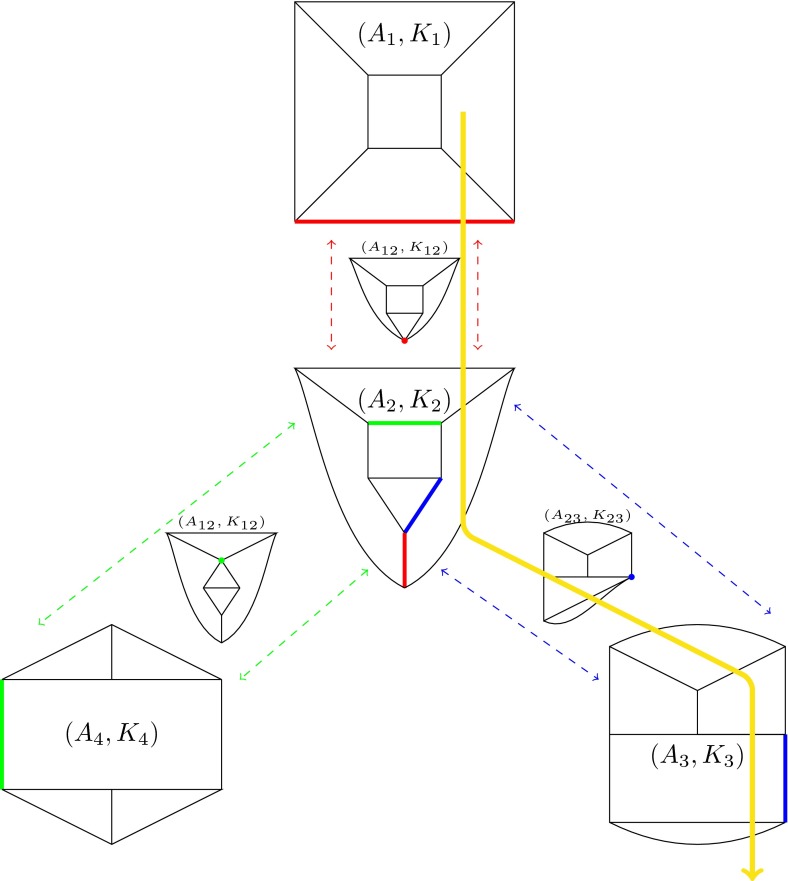
Each pair of graphs $A_i$ and $A_j$ (connected by *dashed arrows*) are related by a single Whitehead move on the colored edges. If these graphs are given consistent labels, then polytopes $K_1,K_2,K_3,K_4$ can be associated with $A_1,A_2,A_3,A_4$, respectively. Each of these graphs have common facets corresponding to their common contracted graphs (smaller intermediate graphs between *dashed arrows*). The *yellow line* represents one possible migration history for a group of six populations whose migration pattern is initially represented by the graph $A_1$, and which subsequently evolve through $A_{12}$, $A_2$ and $A_{23}$ before reaching $A_3$. This particular migration history can then continue evolving via graphs not represented in this diagram

**Fig. 9:**
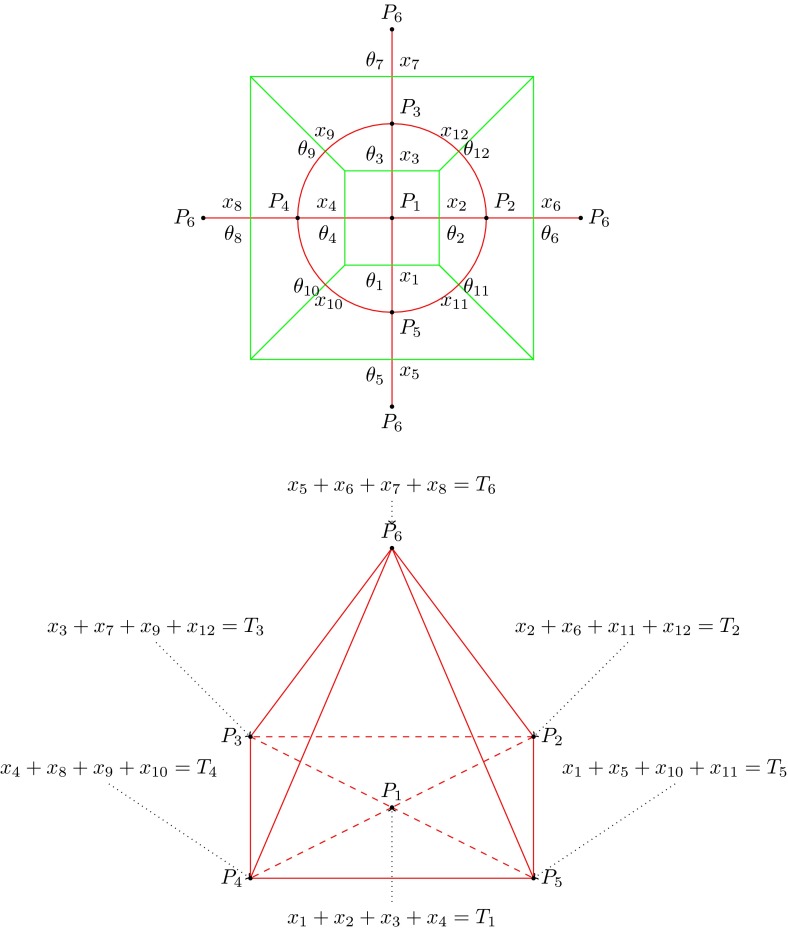
This example shows how a system of equations associated with a labeled Voronoi diagram can be constructed. The graph $G$ (*top*, *green*) represents the Voronoi diagram corresponding to six populations, which are indicated by the vertices $P_1, \ldots , P_6$. By definition, the sum of $x_i$ on each corner must equal $N_i$, as represented in the dual graph (*top and bottom*, *red*) using the variables $x_i$ or equivalently $\theta _i=1-x_i$. The variables $\theta _i$ can be interpreted as intersection angles between the family of circles determined by the vertex of the *triangles in red*. The *lower diagram*, an alternative representation of the *red graph* in the upper diagram (by linking the four $P_6$ vertices), has an equation associated with each vertex, following the definition of the migration pattern

This process is illustrated in Fig. 8. An arbitrary edge labeling $l$ is chosen for graph $A_1$, which determines a system of equations whose solution set is the polytope $K_1$ (as represented in Fig. 9). The central graph $A_2$ is obtained from $A_1$ by a Whitehead move on any of its edges. In this example, we randomly select the lower edge (red) to produce $A_2$, so that the labeling of $A_2$ is inherited from the labeling of $A_1$. As a result, the polytope $K_2$ can be associated uniquely with $A_2$. Exactly two new graphs, $A_3$ and $A_4$, can be obtained from $A_2$ by Whitehead moves. By choosing the labeling on these graphs by Whitehead moves on the blue and green edges, respectively, the polytopes $K_3$ and $K_4$ can be uniquely associated with $A_3$ and $A_4$. When this process is continued by performing all possible Whitehead moves, the polytope complex $\mathfrak {K}=\mathfrak {K}(n,T,l)$ is constructed—i.e., the union of all polytopes that arise through the process described above. The migration history for a set of populations can be encoded by the coordinates of the path on this polytope complex, as long as the number of populations in the system remains fixed. If the number of populations changes, the system jumps to a higher dimensional polytope complex (as described later). The yellow path in Fig. 8 represents the evolving migration pattern of a group of populations. Note, that migration patterns could reach deeper layers of $\mathfrak {K}$ that are not represented in this particular figure (e.g., if the associated graph has more contraction moves than the graph represented by $A_{12}$). Nevertheless, $\mathfrak {K}$ does contain all possible migration scenarios for this system.

At this point, it seems useful to enumerate some properties of $\mathfrak {K}$ that can be deduced from the propositions proved in the Appendix:

1. Each polytope of $\mathfrak {K}$ is associated with a unique cubic graph.
2. If $G_1$ and $G_2$ are cubic graphs connected by a Whitehead move on edge $e$, with associated polytopes $K_1$ and $K_2$, their shared common subgraph $G_{12}$ 7 corresponds to a common face of $K_1$ and $K_2$. This is true because any solution for the linear system of $G_{12}$ can be viewed as a solution for $G_1$ and $G_2$ with the variable corresponding to the contracted edge $e$ set to zero.
3. If a graph $G$ associated with a polytope in $\mathfrak {K}$ is not cubic, its associated polytope can be viewed as a subset of a polytope associated with a cubic graph.
4. The polytope complex $\mathfrak {K}$ depends of the original labeled cubic graph. However, as labeling is related by permutation, corresponding polytopes are related in a similar way.
5. $\mathfrak {K}$ has a finite number of polytopes because the number of cubic graphs, as well as the number of edge labeling possibilities, is finite for a fixed number of vertices.
6. Polytopes in $\mathfrak {K}$ may have common solutions due to the fact that different cubic graphs can give rise to the same contracted graph.
7. The labeling of a graph determines the Euclidean coordinates for a given migration pattern. However, these coordinates are not unique in the complex $\mathfrak {K}$ because a sequence of Whitehead moves can produce several copies of a graph $G$ with different labelings. Nevertheless, considering symmetry of the graphs under translation and rotation, we can say that those coordinates that are symmetric represent the same migration pattern for any given graph.

Additionally, in the space $\mathfrak {K}/sym(S^2)$, where $sym(S^2)$ is the group of symmetric transformations of the two-dimensional sphere generated by translations and rotations (i.e., the Möbius group), every migration pattern is represented uniquely. Note that $\mathfrak {K}$, mathematically a covering space for $\mathfrak {K}/sym(S^2)$, still simplifies the analysis of migration even when a given migration pattern is not uniquely represented on it.

In our view, knowledge of the properties above is important because it provides insight into the dynamics of migration patterns and highlights some considerations that would seem necessary to develop simulation models based on this theory. For example, if we know the migration pattern of a metapopulation at some time $t$ and the associated graph is cubic, we can deduce that the associated graph does not change over short time periods, both future and past, with respect to $t$. We can also deduce that the graph experiences a transition only if the path of the system crosses a face of the associated polytope. An additional application of this theory allows us to simulate metapopulation dynamics by starting with construction of the complex $\mathfrak {K}$. However, we do not need to construct all possible graphs associated with a migration pattern. The properties above instead let us compute the cubic building blocks of $\mathfrak {K}$, which greatly simplifies this task.

Additional mathematical development on the theory of migration patterns seems unnecessary for modeling purposes, and the role of $\mathfrak {K}$ as a feasible computable space where migration histories can be represented as paths is now clear. Only one key point remains: the flexibility to add (or remove) a population from the study system.

### The birth of new populations

We have previously described the evolution of migration patterns using several different perspectives, but always assuming that the number of populations is fixed. In a more realistic setting, the number of populations can vary, and we must consider the effect of introducing a new population into the existing set of populations. We assume that when a new population arises, its location is initially arbitrarily close to the source group (as would be typical in the case of a real population split). However, this assumption does not constrain the applications of this theory because the continuous time model we propose can always be adapted to discrete time periods. Since we represent populations as points, the problem of introducing a new population is equivalent to the problem of introducing a new point $P'_2$ into a set of $n$ points $P=\{P_1,P_2,\ldots , P_n\}$ at time $t_0$, where $P'_2$ is arbitrarily close to $P_2$ (e.g., $lim_{t \rightarrow t_0} P'_2(t)=P_2'(t_0)=P_2(t_0)$). In other words, we need to find the main features of $P'=P \bigcup \{P'_2\}$ under the assumption that $P$ is known. Some relevant observations with respect to the Voronoi decomposition of the sphere are:

1. The Voronoi cell $F_i$ centered at point $P_i$ is the intersection of the entire half sphere $R_{ij}$ determined by the perpendicular bisector $b_{ij}$ of the segment joining the points $P_i$ and $P_j$. However, if $F_i$ is surrounded by cells $F_1,F_2, \ldots , F_m$, then $F_i$ is the intersection of the half sphere $R_{ik}$, where $k \in \{1,2,\ldots , m\}$.
2. If the point $P'_i$ is located in a circular neighborhood $B(P_i,\epsilon )$ with center $P_i$ and radius $\epsilon$, and $P'$ is obtained from $P$ by replacing $P_i$ by $P'_i$, then the Voronoi cell decomposition determined by $P'$ would be close^8^ to the Voronoi cell decomposition determined by $P$ if $\epsilon$ is sufficiently small.

**Fig. 10:**
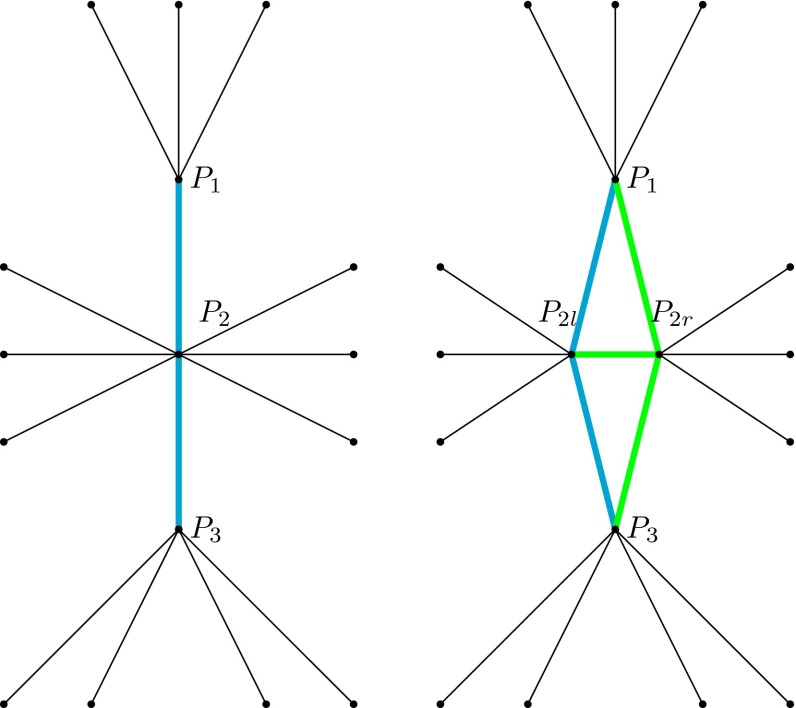
The Delaunay diagram of a set of populations before a population split (*left*), where each *node in black* represents a population. Population 2 then splits into two populations $P_{2l}$ and $P_{2r}$ (*right*). This results in a new Delaunay diagram with three new edges (*green*)

Now assume $P'_2=P_{2r}$ is on the circumference of a very small radius $\epsilon$ and center $P_2=P_{2l}$. The Voronoi cell decomposition determined by $P'$ contains cells of two types: those corresponding to points in $P$ (type I), and two additional cells corresponding to the points $P_{2l}$ and $P_{2r}$ (type II). Since $P_{2l}$ and $P_{2r}$ are very close, type I cells should introduce little variation to adjacent Voronoi cells with respect to $P$. In contrast, the former cell containing $P_2$ has now been divided with one half inside $P_{2l}$ and the other half inside $P_{2r}$. These cells are obtained by the partition produced by the perpendicular bisector of the segment $P_{2l}P_{2r}$ on the former cell containing $P_2$. This splitting process, here described in terms of Voronoi cell decompositions, can also be described in terms of its associated Delaunay cell decomposition (see Fig. 10).

Note that the merger of populations is merely the reverse of the process described above.

## Empirical example

**Fig. 11:**
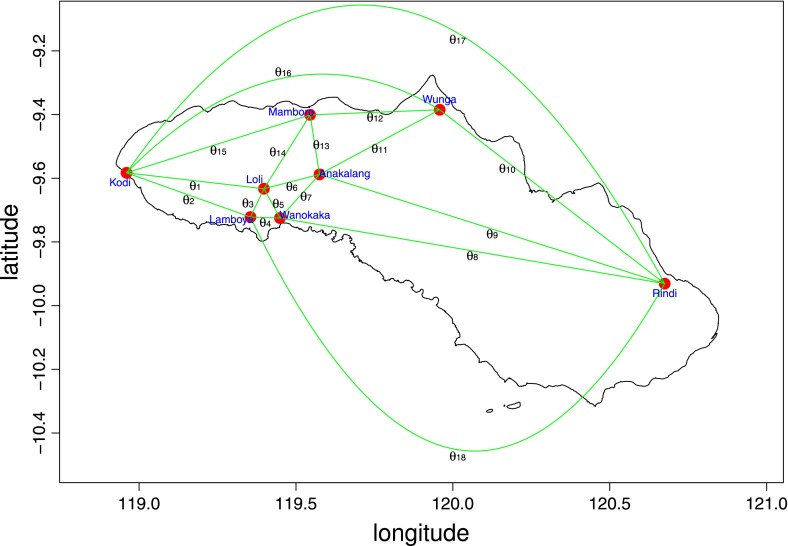
The spherical Delaunay cell decomposition of the sphere for eight populations on the eastern Indonesian island of Sumba

To provide an explicit example of a real-world migration pattern, we chose eight human populations on the eastern Indonesian island of Sumba, for which genetic profiles are well defined (Lansing et al. 2007). The geographical coordinates given in Table 1 let us compute the metapopulation Voronoi diagram, or its equivalent Delaunay cell decomposition, for this system of populations. More exactly, this can be performed by interpreting each population’s latitude and longitude as spherical coordinates of points in the unit sphere, which are then easily converted to cartesian coordinates. Software is available to compute the spherical Delaunay cell decomposition (for example, Zheng 2011). However, since the number of populations here is small, we used a simple R script to enumerate all possible spherical triangles with vertices corresponding to populations $(n = 56)$, and check whether they can form a cell in the metapopulation’s Delaunay cell decomposition of the sphere. We also computed each triangle’s circumcenters,^9^ which allows us to compute the inner angles based in the spherical law of cosines (Gellert et al. 1989). This process leads to the Delaunay cell decomposition of the sphere (Fig. 11), which in our case is a triangulation, and its associated inner triangles (Table 2). To describe the migration pattern for Sumba completely, we also included values for the weights $w_i$ (i.e., measures of gene flow), which in this instance we chose to be the $F_{ST}$ distance between population-level mitochondrial DNA diversity (i.e., a measure of relatedness along the maternal line; Lansing et al. 2007).

**Table 1:** Geographical coordinates for eight Sumba populations

Population	Longitude	Latitude
Kodi	118.960	$-$9.583
Lamboya	119.355	$-$9.722
Loli	119.398	$-$9.633
Wanokaka	119.449	$-$9.725
Mamboro	119.545	$-$9.401
Anakalang	119.575	$-$9.588
Wunga	119.958	$-$9.385
Rindi	120.675	$-$9.931

**Table 2:** Values of the inner angles $\theta _i\; (i=1,2,\ldots , 18)$ using $F_{ST}$ weights

Edge label (i)	1	2	3	4	5	6
$\theta _i$	0.7624459	1.943735	1.875696	2.187413	1.400967	1.189525
$w_i$	0.01113	0.02481	0.06764	0.01297	0.06614	0.0461

Edge label (i)	7	8	9	10	11	12
$\theta _i$	1.681032	1.013773	0.1476047	2.743607	1.337191	2.014021
$w_i$	0.0098	0.00572	0.01157	0.02015	0.04181	0.06872

Edge label (i)	13	14	15	16	17	18
$\theta _i$	1.927833	1.054552	1.286779	0.1883666	2.101859	0.2763413
$w_i$	0.03533	0.04736	0.01528	0.03312	0.00891	0.00536

Using Rivin’s theorem, the sum of inner angles associated with edges incident to a population must sum to $2\pi$. This means that:

$$\begin{aligned} {\left\{ \begin{array}{ll} \theta _1+\theta _2+\theta _{15}+\theta _{16}+\theta _{17}=2\pi ,&{} P_1=Kodi\\ \theta _2+\theta _3+\theta _4+\theta _{18}=2\pi ,&{} P_2=Lamboya\\ \theta _1+\theta _3+\theta _5+\theta _6+\theta _{14}=2\pi ,&{} P_3=Loli\\ \theta _4+\theta _5+\theta _{7}+\theta _{8}=2\pi ,&{} P_4=Wanokaka\\ \theta _{12}+\theta _{13}+\theta _{14}+\theta _{15}=2\pi ,&{} P_5=Mamboro\\ \theta _6+\theta _7+\theta _9+\theta _{11}+\theta _{13}=2\pi ,&{} P_6=Anakalang \\ \theta _{10}+\theta _{11}+\theta _{12}+\theta _{16}+\theta _{17}=2\pi ,&{} P_7=Wunga\\ \theta _8+\theta _9+\theta _{10}+\theta _{18}=2\pi ,&{} P_8=Rindi\\ \end{array}\right. } \end{aligned}$$

which can be verified using data from Table 2.^10^

The total loads of the migration pattern computed in this example are:

$$\begin{aligned} {\left\{ \begin{array}{ll} T_1=w_1+w_2+w_{15}+w_{16}+w_{17}=0.09325, &{} \text {Kodi}\\ T_2=w_2+w_3+w_4+w_{18}=0.11078, &{} \text {Lamboya}\\ T_3=w_1+w_3+w_5+w_6+w_{11}=0.23837, &{} \text {Loli}\\ T_4=w_4+w_5+w_6+w_{7}+w_{8}=0.09463, &{} \text {Wanokaka}\\ T_5=w_{12}+w_{13}+w_{14}+w_{15}=0.16669, &{} \text {Mamboro}\\ T_6=w_6+w_7+w_9+w_{11}+w_{13}=0.14461, &{} \text {Anakalang}\\ T_7=w_{10}+w_{11}+w_{12}+w_{16}+w_{17}=0.17271, &{} \text {Wunga}\\ T_8=w_8+w_9+w_{10}+w_{18}=0.0428, &{} \text {Rindi.}\\ \end{array}\right. } \end{aligned}$$

In this instance, we present the migration pattern corresponding only to the present time. Note that the values $w_i$ carry both geographical and biological information, and can provide considerable insight on subtle population-level dynamics. For instance, the sequence $w_8, w_9, w_{10}, w_{17}, w_{18}$ represents edges connecting Rindi with other populations on Sumba. Consistent with their geographical setting, $w_{17}$ and $w_{18}$ are less likely migration paths and consequently exhibit the smallest values. $w_{10}$, which is intuitively the most likely migration path (along the relatively short distance of the northern coast) shows the largest value. As a second example, edges $w_3$ and $w_5$ link Loli with Lamboya and Wanokaka, respectively. These edges have relatively large values, reflecting close connections between these populations, both geographically and genetically. Curiously, edge $w_4$, which links Lamboya and Wanokaka, is relatively small, hinting that this connection would be worth exploring further within an anthropological setting. The interpretation of $F_{ST}$ (and indeed all other migration metrics) is not straightforward; for a complete review, including alternative interpretations of $F_{ST}$, see Holsinger and Weir (2009).

The question that arises in this context is how to determine the path that populations on Sumba took in the past to reach their present state. While this is a challenging problem, the theory presented here provides a natural analytical framework to address it. However, results must be interpreted in the context of each specific problem. For instance, if we consider a period of time when all migrations were restricted to Sumba, then edges $w_{16}, w_{17}, w_{18}$ would be not considered possible migration pathways, even though they are necessary to fully describe the migration pattern of Sumba’s populations today.

## Discussion and future directions

Population structure has long been known to play a central role in the dynamics of population genetic variation through time. The role of migration has been particularly emphasized by several authors (Hanski and Gilping 1997; Slatkin 1985, 1987). Reconstructing the migration history for a set of populations is crucial for fully understanding the genetics of modern populations. As a step towards this goal, we have presented two equivalent perspectives on the movement of populations (as well as individuals between those populations). These graphs are mirror images of each other, and are based on the graph duality of Voronoi and Delaunay cell decomposition of a two-dimensional sphere. The Voronoi perspective of migration illustrates history as a graph weighted by migration, which suffers continuous deformation through time due to changes in population and individual mobility. Based on contraction and expansion moves in the static case (i.e., a fixed number of populations), splitting or merging populations is also possible. The Delaunay perspective is then represented as dynamic spheres with evolving cell decompositions driven by the addition or deletion of edges (dual to expansion/contraction moves), as well as the addition or deletion of rhomboid structures representing new or merged populations.^11^

**Fig. 12:**
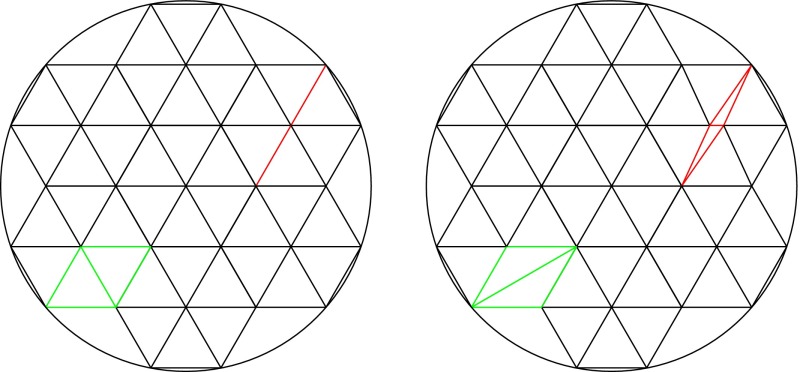
The Delaunay diagram of a group of populations (*left*), each represented by the intersection of three or more edges. The new Delaunay diagram after two transformations in the migration pattern (*right*). The *red segment on the left* is replaced by the *rhomboid structure on the right*, represent the splitting of a population. The *green structure on the left* is replaced by a swap move in the new structure *on the right*. This is equivalent to a Whitehead move on an edge in the corresponding Voronoi decomposition

The migration graph associated with a set of populations can change through time. In Fig. 12, we represent the migration pattern of a large hypothetical set of populations using its Delaunay representation. Each node represents a population, and the migration pattern for this set of populations evolves from left to right. Graphically, this can include two different features, represented by the red and green lines. Changes in these colored structures could have occurred simultaneously or sequentially. Considering these two migration patterns as snapshots of a rotating sphere at two different time points, the observed deformation might result from multiple historical events—changing gene flow, social processes causing the splitting or merging of populations, or the appearance of new physical barriers between populations. The challenge of anthropologists and geneticists is to identify the forces that created the population genetic patterns observed today. Informally, this can be viewed as a rolling ball that suffers deformation under population actions, which in turn produces an unknown path in a known space: $\mathfrak {K}$.

Representing the dynamics of population and individual mobility by paths on the polytope complex $\mathfrak {K}$ simplifies the analysis of migration. In this setting, migration histories, represented as paths in $\mathfrak {K}$, have several analytical advantages: (i) several migration scenarios can be compared by examining the matrices encoding their paths, (ii) different metapopulation systems that are not necessarily related geographically or temporally can be quantitatively compared; and (iii) knowledge of a particular migration pattern can constrain future or past paths in $\mathfrak {K}$, thus reducing the search space in an inferential statistics setting.

The analytical framework presented here allows the number of populations to change as a consequence of splitting and merger events. These splits and mergers could occur sequentially or simultaneously, both of which can be explained using the same construction represented in Fig. 10 (or a straightforward generalization of this construction based on the premise that Voronoi diagrams are largely stable under minor perturbation of the Voronoi cell centers). This also implies that, under significant differences in scale (e.g., regional to global scales), parts of the Voronoi cell decomposition can simply be replaced by a point. This is still a valid representation of the migration system, although now containing less detailed information. For instance, if a set of populations includes groups separated by thousands of kilometers as well as populations separated by only a few kilometers, then close population groups could be modeled at the global scale as a single point. This simplification could be employed to study the dynamics of large scale systems, knowing that local population dynamics could subsequently be re-integrated into the system if required at a later time. This is an especially useful feature, as it can also be employed to handle missing data (which is ubiquitous in most biological datasets).

Further research on the evolution of migration patterns based on a polytope complex is also possible. One productive avenue of research will be optimizing measures of migration to capture the dynamic nature of this process. Although challenging (see Whitlock 1999 for details), there has been substantial progress towards this end in recent years (Hey 2010; Kuhner 2006). Further, there is no obvious standard for how the total weights $T$ assigned to the vertices of a migration graph evolve through time. Although we have previously fixed $T$, it is equally reasonable to consider that $T$ changes through time, thus producing a dynamic polytope complex $\mathfrak {K}$. Hence, the migration history of a set of populations could be viewed as a path in a dynamic $\mathfrak {K}$ with moving walls (i.e., facets of $\mathfrak {K}$).

Further to this idea, a simulation approach based on $\mathfrak {K}$ could be an appropriate starting point to understand changes in population and individual mobility through time. Given known initial and final configurations (as in the ‘Out of Africa’ example used in Sect. 1), Hidden Markov Models with migration patterns as their hidden states might prove a useful way to determine the most likely migration path between two (or more) graph topologies. Frameworks such as these would be radically different to traditional gene lineage based simulators, like SPLATCHE (Ray et al. 1991).

**Fig. 13:**
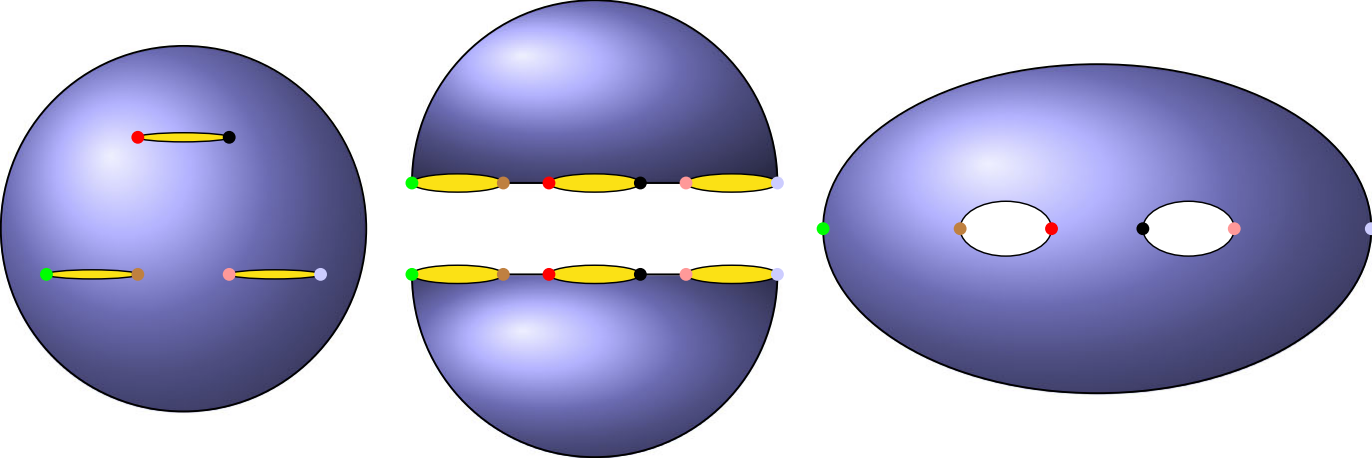
All *points of the same color* represent the same population. A *marked sphere* has been assigned to a set of six populations (*left*). By deforming the sphere (*middle*), we obtain the upper part of the middle panel, which is the mirror image of the lower part. Finally, a double torus is constructed by gluing the top and bottom parts together (*right*)

Finally, we note that there is an analogy between migration patterns and Riemann surfaces. This can be recognized by reference to Riemann surfaces of genus 2 (Amaris 2007) (Fig. 13). Whether progression to the level of abstraction needed to employ Riemann surface theory is directly applicable to population genetics remains unclear, but this would provide another possible avenue for future research. Certainly, Riemann surfaces are related to statistical mechanics, which has previously proven useful for inferring past demographic parameters from modern genetic data (Maruvka et al. 2011). Regardless, studying population dynamics from the perspective of graph theory seems a potentially rich field to infer the impact of mobility on the genes carried by humans (and other populations) around the world.

## Figures and Tables

**Fig. 14:**
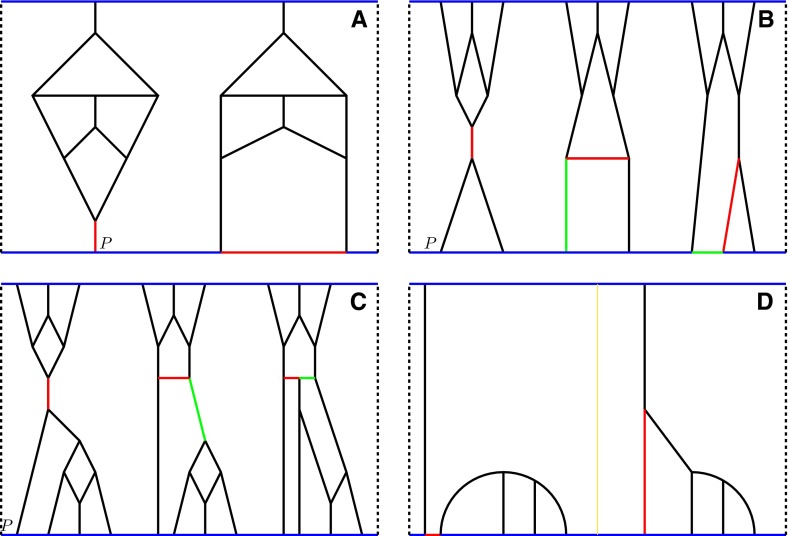
The *four boxes* **A**–**D** represent graphs that connect the top and bottom *blue lines* and are linked by Whitehead moves on *colored edges*. These transformations reduce the number of edges on any path connecting the *top and bottom blue lines*. In **D**, the graphs on the *left and right sides of the yellow line* are connected by a Whitehead move on the *red edge*. This transformation produces a graph that contains at minimum a path joining the top and bottom lines

**Fig. 15:**
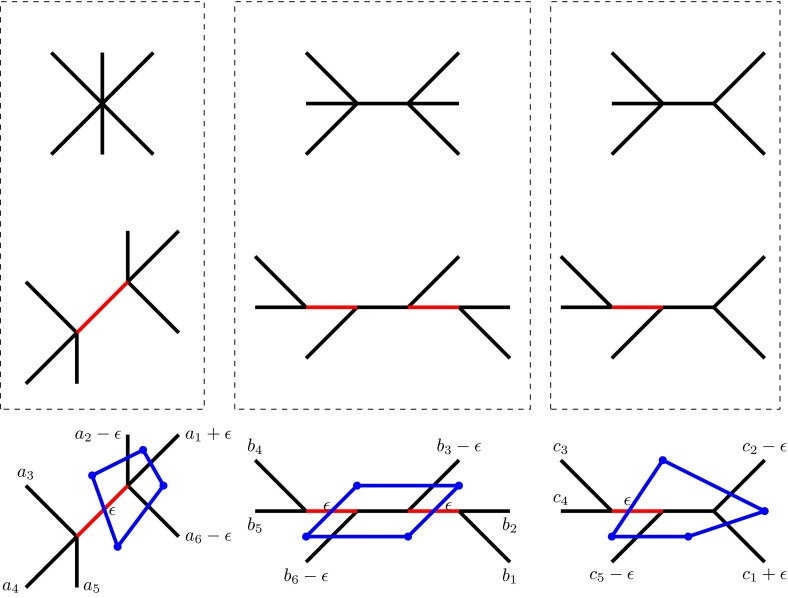
From *left to right*, the *upper dashed boxes* represent expansion transformations of $3$-type, $(2,2)$-type and $(2,0)$-type, respectively. The *blue paths on the bottom row* represent closed paths on the dual of each graph, which allows the definition of an $\epsilon$-modified solution for each expanded graph

## Appendix

Here, we prove the key propositions that underpin the theory presented in this paper. Propositions have been divided into two categories: those related to the combinatorics of $CombMS(n)$, and those related to the polytope complex.

### Combinatorics

The following property of $CombMS(n)$ is known, but as we could not find an explicit reference to it, we provide its proof below.

#### **Proposition 1**

If $G_1$ and $G_2$ are two cubic migration graphs in $CombMS(n)$, then a sequence of Whitehead moves $Wh_{e_1},Wh_{e_2}, \ldots , Wh_{e_k}$ exists such that

$$\begin{aligned} Wh_{e_k} \circ Wh_{e_{k-1}} \circ \ldots \circ Wh_{e_1} (G_1)=G_2 \end{aligned}$$

#### *Proof*

Since $CombMS(i)$ for $i=1,2,3$ has a single element, we can assume $n \ge 4$. To prove our claim for $n \ge 4$, it is sufficient to show that a cubic graph $G$ in $CombMS(n)$ is connected by Whitehead moves to the circular wheel graph $CL_{n}$, as described previously.

We can say that a cubic graph $G$ has two disjoint faces $T$ (top face) and $B$ (bottom face) (Fig. 14) since the only case that does not satisfy this property corresponds to the edges of tetrahedra, which can be transformed by a single Whitehead move to a graph with two disjoint faces.

To complete the proof, it is sufficient to prove that every path^12^ starting at a vertex $\widehat{P}$ of $T$ and ending in a vertex $P$ of $B$ with length greater than one can be shortened by a Whitehead move, while not increasing the length of any other path.

To show this, observe that any point $P$ on the bottom face can be classified according to boxes $A$–$D$ in Fig. 14. In box A, all non-intersecting paths starting at $P$ end in $T$. In box B, a non-intersecting path of length two ends in $B$. In box C, a non-intersecting path of length greater than two ends in $B$. Figure 14 shows that by applying one or two Whitehead moves, the paths joining $B$ with $T$ can be made shorter.

By finding all points in $B$ that can be classified using boxes $A$, $B$ or $C$, and applying their corresponding Whitehead moves, we produce a graph that satisfies the requirement that every path joining $B$ with $T$ has length one. This graph is not necessarily $CL_n$, since it could have paths joining $B$ or $T$ with themselves. However, as described in box $D$, Whitehead moves can be applied to continue the transformation using scenarios $A$, $B$ or $C$ to eventually produce a graph in which all paths of length one connect $T$ and $B$, which is then necessarily $CL_n$.

### Voronoi linear systems

This subsection focuses on properties of the two polytope complexes introduced in the main text. These polytopes are based in two linear systems—geographical and genetic—associated with a graph $G$. Geographical linear systems are obtained by meeting the conditions of Rivin’s theorem (see Theorem 1) for $G$. They comprise a set of equalities for each face of $G$, and one inequality for each loop of $G$. Genetic linear systems, as given by our definition of migration patterns, are linear systems of equations, with one equation for each face of the graph $G$. Some properties of geographical and genetic linear systems can be studied simultaneously by using the more general concept of Voronoi linear systems, which we introduce below. In all of the following propositions, we assume that the linear systems are either geographical or genetic.

First we introduce some useful definitions:

1. A labeling of a graph $G$ is a one to one function $l:E(G) \rightarrow \{1,2,\ldots , card(E(G))\}$, where $E(G)$ and $card(E(G))$ are the set of edges of $G$ and its cardinal.
2. A non-negative face assignment of a Voronoi cell decomposition $\mathcal {D}$ is a one to one function $\sigma : F(\mathcal {D}) \rightarrow \mathbb {R}^{+}_0$, where $F(\mathcal {D})$ is the set of faces (or 2-cells) of the cell decomposition $\mathcal {D}$.
3. The linear equation system $\mathcal {E}=\mathcal {E}(\mathcal {D},l,\sigma )$ associated with the Voronoi cell decomposition $\mathcal {D}$ with non-negative face assignment $\sigma$ and labeling $l$ is given by:
  $$\begin{aligned} \mathcal {E}: \sum _{e \in F} x_{l(e)}=\sigma (f) \end{aligned}$$
  for all faces $f \in F(\mathcal {D}$).
4. A constraint system is a set of inequalities in the variables $x_{l(e)}$ (possibly empty) denoted by $\mathcal {C}$. The compaction of $\mathcal {C}$, $\mathcal {\bar{C}}$, is by definition obtained by replacing any strict inequality $<,>$ of $\mathcal {C}$ respectively by $\le , \ge$.
5. By definition, the Voronoi linear system $\mathcal {L}(\mathcal {D},\mathcal {C})$ associated with $\mathcal {D},\mathcal {C}, l, \sigma$ is given by $(\mathcal {E},\mathcal {C})$. Hence a Voronoi linear system has a linear equation system $\mathcal {E}$ and a constraint system $\mathcal {C}$. If we substitute $\mathcal {C}$ with $\mathcal {\bar{C}}$, we call the resulting linear system a compacted Voronoi linear system.

Whitehead moves, which were introduced as transformations on cubic graphs transforming a graph $G$ into a graph $G'$ for a given edge $\widehat{e}$, can be viewed as a process that changes a Voronoi linear system $\mathcal {L}$ to a new Voronoi linear system $\mathcal {L'}$. Indeed, since a Whitehead move in our study can be considered as a continuous deformation process of a graph embedded in the sphere,^13^ the cell decomposition $\mathcal {D}$ is transformed into a new cell decomposition of the sphere $\mathcal {D'}$ having a Voronoi diagram $G'$, which inherits a labeling from the Voronoi diagram $G$ of $\mathcal {D}$, and each face $f'$ of $\mathcal {D'}$ corresponds uniquely to a face $f$ of $\mathcal {D}$. This also allows Whitehead moves to be viewed as transformations acting on a set of faces.

Whitehead moves act on linear equation systems by changing the linear equation system $\mathcal {E}$ to $\mathcal {E'}$, which is defined by

$$\begin{aligned} \mathcal {E'}: \sum _{e' \in f'} x_{l(\alpha ^{-1}(e'))}=\sigma (\beta ^{-1}(f')) \end{aligned}$$

for all faces $f' \in F(\mathcal {D'}$), where $\alpha :E(D)\rightarrow E(D')$ and $\beta :F(D)\rightarrow F(D')$ are bijections obtained by a deformation of the cell decomposition $\mathcal {D}$ into $\mathcal {D'}$. The linear equation system $\mathcal {E'}$ has equations that are identical to the ones in $\mathcal {E}$ for faces that were obtained from faces of $\mathcal {D}$, which were not incident to the edge $\widehat{e}$. If the face $f'$ comes from a face that either ‘loses’ or ‘gains’ an edge, the corresponding variable is deleted or added in the equation associated with $f'$.

For further analyze the action of Voronoi linear systems, we next introduce the notion of an edge-contraction coherent collection of constraint systems, which is necessary to construct a polytope complex associated with a metapopulation system.

#### **Definition 4**

*(Edge-contraction coherent collection of constraint systems)* Let $\mathbb {L}=(\mathcal {E}_i,\mathcal {C}_i)_{i \in I}$ be a collection of Voronoi linear systems with an associated collection of Voronoi diagrams $(G_i)_{i \in I}$. We say that $\mathcal {C}=(\mathcal {C}_i)_{i \in I}$ is an edge-contraction coherent collection of constraint systems, if for all of its pairs of constraint systems $\mathcal {C}_i$ and $\mathcal {C}_{i'}$, such that $G_{i'}$ is obtained by a contraction move from $G_i$ on its edge $e$, we can get $\mathcal {C}_{i'}$ simply by deleting all the variables associated with edge $e$ in $\mathcal {C}_{i}$. In this case, we will say that $\mathbb {L}$ is a coherent collection of Voronoi linear systems.

Since we need to prove several properties of Voronoi linear systems related to polytope theory, we provide two known equivalent definitions of polytope below.

#### **Definition 5**

By definition, the convex hull of a finite set of points $K$ in $\mathbb {R}^d$ is a polytope. Hence,

$$\begin{aligned} K=\left\{ \sum _{1=1}^{i=n}\lambda _ix^i|\lambda _i \ge 0, \sum _{i=1}^{i=n} \lambda _i=1\right\} \end{aligned}$$

for a subset $X=\{x^i|i \in I\}$ of $\mathbb {R}^d$.

#### **Definition 6**

$K$ is a polytope if $K$ is a bounded solution set of a finite system of linear inequalities:

$$\begin{aligned} K=\{x \in \mathbb {R}^d | a_i^{T }x \le b_i \text { for } 1 \le i \le m \} \end{aligned}$$

where $A$ is a real matrix of dimension $m \times d$ and $b \in \mathbb {R}^d$.

The solution set of the compacted Voronoi linear system $L(\mathcal {D},\mathcal {C})=(\mathcal {E},\mathcal {\bar{C}})$, $K=K(\mathcal {E},\mathcal {\bar{C}})$, is a polytope, as we prove next.

#### **Proposition 2**

If $K=K(\mathcal {E},\mathcal {\bar{C}})$ is the solution set of a compacted Voronoi linear system, then $K$ is a polytope.

#### *Proof*

Let $\mathcal {E}=E(\mathcal {D},l,\sigma )$ and let $n$ be the number of edges in $E(\mathcal {D})$. For every edge $e_i$, $i \in \{1,2,\ldots ,n\}$, of $E(\mathcal {D})$, choose a face of $f_i \in F(\mathcal {D})$. If $x=(x_i)_{i=1}^{n}$ is in $K$,

$$\begin{aligned} x_i \le \sum _{e \in f_i} x_{l(e)}=\sigma (f_i) \le Max(\sigma (f_j)|j \in \{1,2,\ldots ,n\}) \end{aligned}$$

then $|x|^2 \le n^2 Max^2(\sigma (f_j)|j \in \{1,2,\ldots ,n\})$. Therefore, $x$ is bounded, and hence by definition, $K$ is a polytope.

#### **Definition 7**

Given a Voronoi linear system $(\mathcal {E},\mathcal {C})$, we denote the collection of all realizable graphs in $H \in CombMS(n)$ by $\varPsi (n)$, the collection of all polytopes associated with any graph $H \in \varPsi (n)$ by $\varOmega (n)$, and define $\varGamma (n)=\{(G,\theta ):G \in \varPsi (n), \theta \in \varPsi (n)\}$. If $\mathcal {C}=\emptyset$, we denote the above collection by $\varPsi _0(n)$, $\varOmega _0(n)$ and $\varGamma _0(n)$.

#### **Definition 8**

1. A generalized Whitehead move on a graph $G$ is the combination of a contraction and expansion move on G.
2. A chain of graphs in $CombMS(n)$ is a sequence of graphs $H=(H_1,H_2, \ldots , H_m)$ in $CombMS(n)$ such that $H_i$ and $H_{i+1}$ are connected by a contraction, expansion or generalized Whitehead move.
3. The depth of a sequence of graphs $H=(H_1,H_2, \ldots , H_m)$ in $CombMS(n)$ is given by $depth(H)=Max(depth(H_i): i \in 1,\ldots , m)$.
4. A chain $S=(S_1,S_2, \ldots , S_m)$ is a lifting of $H=(H_1,H_2, \ldots , H_k)$ if $depth(H')<depth(H)$ and for each $i=1,2,\ldots , k$, there exists $j(i) \in \{1,2,\ldots , m\}$ such that $H_i=S_{j(i)}$ or $S_{j(i)}$ can be obtained by an expansion move from $H_i$. Observe that in this definition $j$ is not necessarily one to one.

#### **Proposition 3**

If $G_1, G_2 \in \varPsi _0(n)$, then a sequence $m_1,m_2, \ldots , m_k$ exists where $m_i$ is a contraction or expansion move, such that $H_i=m_1 \circ m_2 \circ \ldots \circ m_i(G_1) \in \varPsi _0(n)$ for $i=1,2, \ldots , k$ and $H_k(G_1)=G_2$. In other words, $\varPsi _0(n)$ is connected.

#### *Proof*

It is sufficient to prove that every connected component of $\varPsi _0(n)$ includes the graph $C_n$, as defined previously (represented by $C_{10}$ on Fig. 5), and that it is a realizable graph, which admits as a solution the n-dimensional vector with all entries equal to $\pi$, $\theta ^{[0]}$, which can be proved by contradiction as follows:

(i) Let $G_1$ be a graph in $\varPsi _0(n)$, and $\varPsi _{01}$, $\varOmega _{01}$ the connected components of $\varPsi _0(n)$ and $\varOmega _0(n)$ corresponding to all realizable graphs that are connected to $G_1$, respectively.
(ii) Define the function $h:\varPsi _{01} \rightarrow \mathbb {R}_0^{+}$ by
  $$\begin{aligned} h(G)=min\{i:m_1 \circ m_2 \circ \ldots \circ m_i(C_i)=G,\theta \in \varOmega _{01} \text { corresponding to G}\} \end{aligned}$$
  $h$ is well defined since the number of sequences that join $G$ with $C_n$, by contraction or expansion moves, is finite.
(iii) Let $G_2$ be such that $h(G_2)$ is a minimum and choose $k^{[2]}$ such that $h(G^{[2]})=k^{[2]}$.
(iv) If $G_2$ has a vertex of even valence $P$, choose an even closed loop around $P$, in the dual graph of $G_2$, $P_1,P_2, \ldots , P_{2m}$ and assume the labels corresponding to the edges of $G_2$ that this loop crosses are $1,2, \ldots , 2m$. Then, we can define an $\epsilon$-modified solution of $G_2$, $\theta ^{[2,\epsilon ]}$ by $\theta ^{[2,\epsilon ]}_i=\theta ^{[2]}_i$ for $i \ne 1,2, \ldots , 2m$ and $\theta ^{[2,\epsilon ]}_i=\theta ^{[2]}_i+{(-1)}^i \epsilon$ for $i \ne 1,2, \ldots , 2m$. Without loss of generality, suppose that $\theta _1^{[2]}=min\{\theta _i^{[2]}: i=1,2,\ldots , 2m\}$. Taking $\epsilon =\theta _1$, we get a new graph $G^{[3]}$ that is obtained by a contraction move on edge $e_1$ such that $h(G^{[3]})<h(G^{[2]})$, which is a contradiction. Notice that if $G^{[2]} \ne C_n$, $e_1$ cannot be incident to a bigon (a polygon with two edges and two vertices) because this would imply that either the neighboring faces of the bigon are a bigon themselves and have minimal edges incident to them (from the fact that the sum of $\theta _i$ at each face is $2\pi$), and repeating this argument, all faces incident to $P$ are bigon. In this case, we will show that $G^{[2]}=C_n$, which proves our claim.
(v) If $G^{[2]}$ does not have any vertex of even valence, we can choose any edge joining vertices $P_1$ and $P_2$ and define an even loop around them. Applying a similar argument to that above, we can conclude that $G^{[2]}=C_n$, which proves our claim.

We believe that $\varPsi (n)$ is also connected. However, although everything said in the argument above is true for $\varPsi (n)$, we do not prove the constraint inequalities of Rivin’s theorem, which may fail for a dual graph loop that passes through the edges whose solutions are effectively being changed. Hence, we leave the connectivity of $\varPsi (n)$ as a conjecture, believing that a modification or extension of the proof above is possible.

#### **Proposition 4**

Every chain $H$ in $\varPsi (n)$ has a lifting $H$ in $\varPsi (n)$.

#### *Proof*

Let $H=(H_1,H_2, \ldots , H_m)$ be a chain in $\varPsi (n)$ with $k=depth(H)$. If $depth(H_i)<k$, define $H'_i=H_i$. Otherwise, at least one of the following conditions is satisfied:

- $H_i$ has a vertex $P$ with $degree(P)\ge 5$.
- $H_i$ has an edge $e=\bar{RS}$ where $degree(R)=2$ and $degree(S)=3$ (or vice versa).
- $H_i$ has an edge $e=\bar{RS}$ where $degree(R)=degree(S)=4$.

We choose one of the above, which is satisfied, and apply an expansion move of $3$-type, $(2,0)$-type or $(2,2)$-type as illustrated in Fig. 15 to obtain $H'_i$, which satisfies $depth(H'_i)=depth(H)-1$.

Two consecutive elements $H'_i$ and $H'_{i+1}$ of the new sequence $H$ trivially satisfy the two conditions of the definition of a lifting if $H'_i=H_i$ and $H'_{i+1}=H_{i+1}$. Otherwise, if $H'_i=H_i$ or $H'_{i+1}=H_{i+1}$, $H'_i$ and $H'_{i_+1}$ are connected by a generalized Whitehead move, and assuming that $H'_{i+1}=H_{i+1}$, we can say that $H'_i$ is realizable. This is because we can build an $\epsilon$-modified solution $(a'_i)$, $(b'_i)$ or $(c'_i)$ defined from the respective solution $(a_i)$, $(b_i)$ or $(c_i)$ of $H_i$ according to the type of expansion transformation used, as suggested by the diagram at the bottom of Fig. 15. We define $(a'_i)$, $(b'_i)$ or $(c'_i)$ by keeping the same values as in $(a_i)$, $(b_i)$ or $(c_i)$, except for the edges that are crossed by the blue loops, which are modified by $a \pm \epsilon$. This defines a solution of the Voronoi system of $H'_i$ for a sufficiently small $\epsilon$ because its equation system is satisfied as the signs of $\epsilon$ are opposite for each node in the dual graph of $H'_i$, which has two incident blue edges. Also note that the constraint inequalities of $H'_i$ are also satisfied.

## Footnotes

1 Homeomorphism indicates that a function exists with continuous inverse of the sphere that transforms the first graph into the second.

2 We allow a graph to be a hypergraph—that is, it can contain multiple edges between two vertices.

3 Here, we specifically have in mind genetic measures such as $F_{ST}$, although alternative metrics, including estimates of migration, either direct or inferred (Hey 2010), are equally well suited.

4 Note that $CombMS(n)$ does not depend on $T$.

5 Edge labeling is a one-to-one assignment of the numbers $1,2, \ldots , m$ to the set of edges.

6 At the intermediate step of a Whitehead move, a label is momentarily lost whenever an edge is deleted. We define that, in the expanding step, the new edge is given the label of the old lost edge.

7 The common subgraph $G_{12}$ is obtained in the intermediate step of the Whitehead move connecting $G_1$ and $G_2$.

8 Close in the sense that the edges of $P$ could be covered by circles of some small radius $\epsilon$, such that the edges of $P'$ are contained in the union of this cover.

9 Each spherical triangle has two circumcenters, which are the antipodes of each other.

10 Note that our data also fulfills the second condition of Rivin’s theorem.

11 A rhomboid structure is represented by the union of blue and green edges in Fig. 10.
